# Squamous cell carcinoma and Crohn's disease: a sometimes-challenging diagnosis

**DOI:** 10.2144/fsoa-2023-0109

**Published:** 2024-05-15

**Authors:** Mona Boudabbous, Baha Barkia, Wiem Feki, Héla Gdoura, Lassad Chtourou, Manel Moalla, Leila Mnif, Ali Amouri, Zeinab Mnif, Nabil Tahri

**Affiliations:** 1 Gastroenterology Department, Hédi Chaker Hospital, Sfax, 3000, Tunisia; 2 Radiology Department, Hédi Chaker Hospital, Sfax, 3000, Tunisia; 3 Medecin Sfax University, Sfax university, 3000, Tunisia

**Keywords:** combination therapy, inflammatory bowel disease, squamous cell carcinoma, thiopurines

## Abstract

**Aim:** Non-melanoma skin cancers are more common in people with inflammatory bowel disease. However, these tumors can rarely mimic a cutaneous manifestation of the disease, which delays diagnosis and clouds prognosis. **Observation:** A 35-year-old man with stenosing and fistulizing ileocolic Crohn's disease developed squamous cell carcinoma mimicking a groin fold abscess. After surgical drainage of the abscess, despite antibiotics and therapy combining azathioprine with infliximab, the abscess has recurred. Biopsies revealed a cutaneous squamous cell carcinoma. Palliative radiotherapy-chemotherapy was initiated, but the patient died after 3 months. **Conclusion:** This observation illustrates the increased risk of non-melanoma skin cancers in inflammatory bowel disease patients, particularly those exposed to thiopurines, and the value of diagnosing them at an early stage.

## Abstract (plain language summary)

Clinicians should be aware of the risk of skin neoplasia in patients with inflammatory bowel disease (IBD) on long-term azathioprine treatment.

Malignancy should be considered in patients with IBD longstanding peri-anal disease especially in the case of atypical location or appearance.

Where squamous cell carcinoma develops in patients with Crohn's disease, there is often a delay in appropriate medical management due to a low level of clinical suspicion of malignancy.

It is important for clinicians to be cautious when treating immunocompromised patients with skin lesions, and early consideration of biopsy is recommended.

Early diagnosis and timely intervention are essential in the management of squamous cell carcinoma, as a combination of surgical and oncological approaches can potentially lead to a cure.

Non-melanocytic skin cancers, including basal cell carcinoma and squamous cell carcinoma, are very common in the general population. However, people with inflammatory bowel disease (IBD), regardless of treatment, have a higher incidence of these skin cancers, with an increased risk ranging from 20 to 50% [1,2].

The use of thiopurine-based drugs, such as azathioprine, has been associated with a twofold increase in the risk of developing skin cancer in IBD patients [3,4]. Whether this risk reverses when treatment is stopped remains uncertain [2,5].

We report the case of a patient followed for Crohn's disease (CD) who developed squamous cell carcinoma with a fatal outcome. The importance of this case lies in the delayed diagnosis, which limited therapeutic possibilities and had severe consequences on prognosis. Then, malignancy should be considered in patients with IBD longstanding peri-anal disease especially in the case of atypical location or appearance.

## Observations

We report the case of a 35-year-old Caucasian man with stenosing and fistulizing ileocolic CD and ano-perineal manifestations of complex fistulas. As a result of complications related to his disease, the patient underwent a segmental resection of approximately 30 cm of the diseased small intestine, with subsequent ileo-caecal anastomosis.

The patient was initially prescribed azathioprine as a maintenance treatment in 2006. However, the disease progression was characterized by recurrent relapses, necessitating the administration of intravenous corticosteroids. Based on the 6TGN assay, the patient's thiopurine metabolite levels were found to be within the therapeutic range.

**Figure 1.**
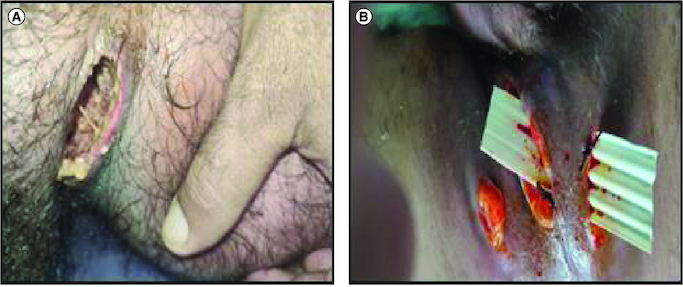
Initial appearance of the lesion in preoperative (A) and postoperative conditions (B).

In 2017, due to the ineffectiveness of azathioprine, treatment with infliximab was initiated as part of combination therapy, following a thorough pre-therapeutic workup that included a normal dermatological examination. In 2020, azathioprine was discontinued due to the discovery of profound thrombocytopenia. Anti-tumor necrosis factor therapy was initially administered and subsequently continued as a maintenance treatment with optimized dosing and duration. The patient received a total of 51 courses of infliximab. During the course of treatment, in 2022, the patient developed a swelling in the left groin fold. A pelvic CT scan revealed a multicompartment collection in the left inguinal canal. The collection consisted of heterogeneous hydroaerous content, primarily liquid, with a poorly defined boundary. After injection of a contrast agent, the wall of the collection showed enhanced visualization. The dimensions of the collection were approximately 6.5 cm in the coronal plane and extended about 6.4 cm in the sagittal plane. It was in close proximity to the homolateral psoas and Sartorius muscles. Initially, a flattening procedure was performed locally (Figure 1A). However, after 3 months, due to non-healing and the presence of purulent discharge, the patient underwent surgical drainage (Figure 1B). Antibiotic therapy was initiated, leading to favorable progress in the patient's condition.

**Figure 2.**
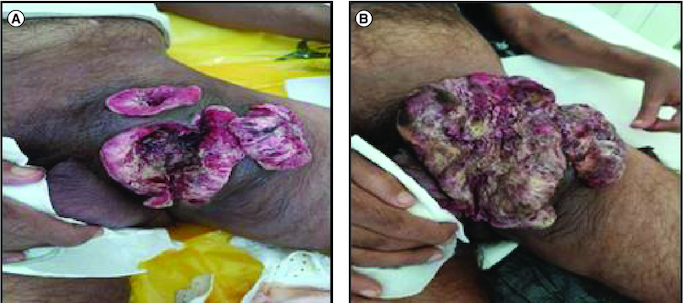
Rapid tumor evolution from September 2022 (A) to December 2023 (B).

**Figure 3.**
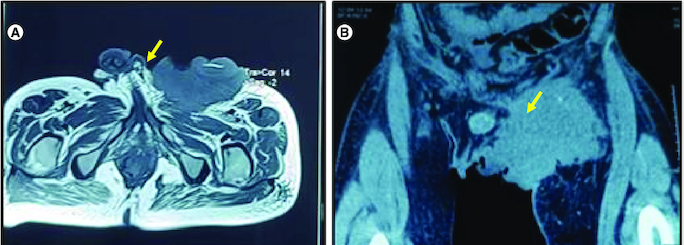
Radiological appearance of the tumor. **(A)** MRI: Axial T2-weighted section centered on the pelvic region: large left inguinal mass in T2 hypoposignal (yellow arrow). **(B)** CT scan of the tumor coronal reconstruction without injection of contrast medium: left inguinal mass spontaneously hypodense (yellow arrow).

**Figure 4.**
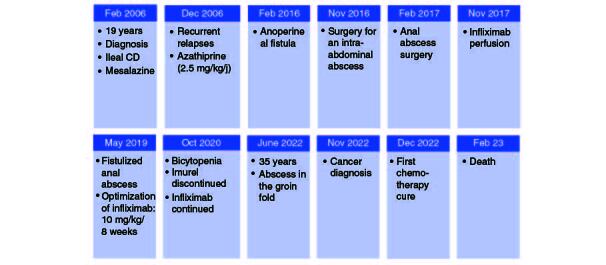
Clinical history of the patient. CD: Crohn's disease.

During the healing process of the post-drainage wound in the groin fold abscess, an hypertrophic budding scar was observed (Figure 2A). To further investigate the nature of the scar, biopsies were performed. Biopsy analysis showed cutaneous squamous cell carcinoma. Pathological examination of the biopsies revealed a well-differentiated invasive squamous cell proliferation. It is composed of clumps of squamous cells showing pronounced atypias, nuclear monstruosities and a poorly developed dermoplastic stroma. The course was characterized by rapid progression of the disease (Figure 2B). A pelvic RMI and CT scan was performed, revealing a left inguinal tissue mass with heterogeneous density and enhancement. The mass displayed irregularly contoured necrotic areas and had increased in size compared with the initial imaging, measuring approximately 11 cm in the coronal plane, 10.5 cm in the sagittal plane, and 8.2 cm in the axial plane. It invaded the left pectineal muscle without evidence of bone lysis opposite the left iliopubic branch. Additionally, the mass invaded the homolateral obturator externus, adductor longus and gracilis muscles. It exhibited close contact with the left common femoral vein, with a conference angle of less than 180 degrees and a loss of the safety fat border. The mass also showed proximity to the structures of the left inguinal canal and corpora cavernosa, causing displacement of these structures toward the right side. Multiple bilateral inguinal adenopathies with small infracentimeter axis and an osteolytic lesion of the right iliac wing were also noted in association with the mass (Figure 3). The decision was palliative radiotherapy–chemotherapy. Although pembrolizuma and cemiplimab are currently indicated as first-line treatments for squamous cell carcinomas, these compounds were not available in our country. The patient was therefore given one course of cisplatin-based chemotherapy and there has been a slight clinical improvement in the local condition, but he died a month later due to tumor progression. The clinical history of the patient is represented in Figure 4.

## Discussion

Squamous cell carcinoma (SCC) is a skin cancer that develops from the malignant transformation of squamous cells, which are flat, thin cells located in the outer layer of the skin [6]. One possible explanation for the increased incidence of skin cancer in patients taking azathioprine is the increased sensitivity to UVA light that the drug can induce [7]. Azathioprine is known to increase photosensitivity, resulting in an exaggerated response to UVA light and potentially contributing to sunburn and skin lesions. For this reason, it is essential that patients taking azathioprine are aware of this increased risk of skin cancer and take the necessary precautions to minimize sun exposure. This includes wearing protective clothing, using sunscreen with a high sun protection factor and avoiding direct exposure to the sun during peak hours. Regular skin examinations and early detection of any suspicious skin lesions are also important for monitoring and managing the risk of skin cancer.

The risk of skin cancer, including SCC, is influenced by factors such as IBD and its treatment. However, several other factors [8] contribute to the development of CSC in these patients. These include poor wound healing, persistent cell turnover in areas affected by inflammation and trauma, chronic immunosuppression and a reduced immune response to tumor cells present in the scar tissue [^8-10^].

Perianal fistulizing CD is a very disabling condition with poor quality of life. Patients with perianal fistulizing CD are also at risk of perianal fistula-related SCC. Cancer arising at the site of a chronic perianal fistula is rare in patients with CD and there is a paucity of data regarding its incidence, diagnosis and management. The incidence rate of SCC related to perianal fistula is very low (<1%). Prognosis is poor. Colorectal disease, chronic perianal disease and HPV infection are possible risk factors. As with our patient, fistula-related carcinoma in CD can be very difficult to diagnose. Examination may be limited by pain, strictures and induration of the perianal tissues. HPV is an important risk factor with a particular carcinogenesis mechanism. MRI can help clinicians in diagnosis. Examination under anesthesia is highly recommended when findings, a change in symptoms, or simply long-standing disease in the perineum are present [6]. In our patient, the unusual location of the fistulized abscess in the groin crease, never described in the literature, should raise suspicion of malignant pathology.

In cases, such as our patient's, where squamous cell carcinoma develops in patients with CD, there is often a delay in appropriate medical management due to a low level of clinical suspicion of malignancy. It is important for clinicians to be cautious when treating immunocompromised patients with skin lesions, and early consideration of biopsy is recommended. Early diagnosis and timely intervention are essential in the management of SCC, as a combination of surgical and oncological approaches can potentially lead to a cure [11].

## Conclusion

Skin cancer, including SCC, is an important concern in patients with IBD and its associated treatments. It is crucial that patients diagnosed with IBD take necessary precautions to protect themselves from sun exposure and undergo regular skin examinations. In our patient, the unusual location of the fistulized abscess in the groin crease, never described in the literature, should raise suspicion of malignant pathology. Early diagnosis and timely intervention are essential in the management of squamous cell carcinoma is the only way to save these patients.
